# Assessment of Appropriateness of Antimicrobial Therapy in Resource-Constrained Settings: Development and Piloting of a Novel Tool—AmRAT

**DOI:** 10.3390/antibiotics10020200

**Published:** 2021-02-19

**Authors:** Ashish Kumar Kakkar, Nusrat Shafiq, Neeru Sahni, Ritin Mohindra, Navjot Kaur, Nanda Gamad, Aditi Panditrao, Dimple Kondal, Samir Malhotra, Praveen Kumar M, Rachna Rohilla, Samiksha Bhattacharjee, Ankit Kumar, Ritika Kondel Bhandari, Avaneesh Kumar Pandey, Imraan Rather, Chakrant Mothsara, Cvn Harish, Devaraj Belavagi, Gopal Vishwas

**Affiliations:** 1 Postgraduate Institute of Medical Education and Research, Chandigarh 160012, India; drashishkakkar@gmail.com (A.K.K.); neerunalin@yahoo.com (N.S.); ritin.mohindra@gmail.com (R.M.); nanda.gamad@gmail.com (N.G.); smal.pgi@gmail.com (S.M.); praveenkumarpgiindia@gmail.com (P.K.M); rachna.rohilla20@gmail.com (R.R.); samagmc@gmail.com (S.B.); excludeankit@gmail.com (A.K.); ritikakondal12@gmail.com (R.K.B.); avaneeshkpandey@gmail.com (A.K.P.); ratherimraan@gmail.com (I.R.); chakrant.mothsara@gmail.com (C.M.); cvenhar@gmail.com (C.H.); belavagidevraj779@gmail.com (D.B.); singhgopalvishwas@gmail.com (G.V.); 2 Vardhman Mahavir Medical College & Safdarjung Hospital, New Delhi 110029, India; drnavjotpharma@gmail.com; 3 Adesh Institute of Medical Sciences and Research, Bathinda 151101, India; drpandiboo@gmail.com; 4 Public Health Foundation of India, Gurugram 122002, India; dimple@ccdcindia.org

**Keywords:** antimicrobial prescribing, antimicrobial stewardship, appropriateness of antimicrobial therapy, lower- and middle-income countries

## Abstract

Inappropriate antimicrobial prescribing is considered to be the leading cause of high burden of antimicrobial resistance (AMR) in resource-constrained lower- and middle-income countries. Under its global action plan, the World Health Organization has envisaged tackling the AMR threat through promotion of rational antibiotic use among prescribers. Given the lack of consensus definitions and other associated challenges, we sought to devise and validate an Antimicrobial Rationality Assessment Tool—AmRAT—for standardizing the assessment of appropriateness of antimicrobial prescribing. A consensus algorithm was developed by a multidisciplinary team consisting of intensivists, internal medicine practitioners, clinical pharmacologists, and infectious disease experts. The tool was piloted by 10 raters belonging to three groups of antimicrobial stewardship (AMS) personnel: Master of Pharmacology (M.Sc.) (*n* = 3, group A), Doctor of Medicine (MD) residents (*n* = 3, group B), and DM residents in clinical pharmacology (*n* = 4, group C) using retrospective patient data from 30 audit and feedback forms collected as part of an existing AMS program. Percentage agreement and the kappa (κ) coefficients were used to measure inter-rater agreements amongst themselves and with expert opinion. Sensitivity and specificity estimates were analyzed comparing their assessments against the gold standard. For the overall assessment of rationality, the mean percent agreement with experts was 76.7% for group A, 68.9% for group B, and 77.5% for group C. The kappa values indicated moderate agreement for all raters in group A (κ 0.47–0.57), and fair to moderate in group B (κ 0.22–0.46) as well as group C (κ 0.37–0.60). Sensitivity and specificity for the same were 80% and 68.6%, respectively. Though evaluated by raters with diverse educational background and variable AMS experience in this pilot study, our tool demonstrated high percent agreement and good sensitivity and specificity, assuring confidence in its utility for assessing appropriateness of antimicrobial prescriptions in resource-constrained healthcare environments.

## 1. Introduction

Antimicrobial resistance (AMR) is widely recognized as a growing, complex, and multifaceted public health problem that significantly affects populations in resource-constrained low- and middle-income country settings (LMICs). Currently, nearly 700,000 patients succumb to drug-resistant infections annually across the globe, and it has been estimated that in the absence of urgent and concerted efforts, this figure could increase to 10 million deaths by 2050 [1]. According to recent estimates based on drug sales data across 76 countries, global per capita consumption of “Watch” group antibiotics jumped by 91% compared to an increase of 26% for “Access” group medicines between 2000 and 2015. The increase in Watch group consumption was greater in LMICs as compared to high-income countries (HICs). Alarmingly, more than 90% of LMICs had a decrease in their relative Access-to-Watch antibiotic consumption [2]. Similar concerning trends were observed in a recent multicentric point prevalence survey conducted across India [3]. Over the period of 15 years (2000–2015), global utilization of antibiotics increased by 65% from 21 to 35 billion defined daily doses (DDDs), while the consumption rate increased by nearly 40%, being primarily driven by enhanced usage in LMICs. The antimicrobial utilization in LMICs is rapidly rising to match the rates prevalent in HICs [4]. If the current trends go unabated, the financial consequences could be catastrophic too, forcing 24 million people to extreme poverty by 2030 [1]. In addition, emerging infectious diseases, including those appearing for the first time, those with significantly increased incidence, or diseases that have crossed the species barrier, pose significant threat to human health in terms of morbidity and mortality, as well as adverse social and economic consequences [5,6,7].

Misuse and overuse of antimicrobials have been universally recognized as the key drivers for the emergence of drug-resistant pathogens [8,9,10,11]. Given the significant gap in the pipeline for the development of novel and effective antimicrobials, the only feasible way to increase the longevity of existing drugs is to curb unnecessary prescription and inappropriate consumption of these agents [12]. The World Health Organization has also endorsed the need to preserve existing antimicrobials which is reflected in its slogan for antimicrobial awareness week 2020 [13]. In 2015, the World Health Assembly endorsed a global action plan to address the threat of AMR by reducing the incidence of infectious diseases, enhancing knowledge and understanding of AMR through research and surveillance, enhancing investment for the development of novel interventions, and promoting rational use of antimicrobials [14].

Although universally accepted standardized definitions of appropriate antimicrobial use are lacking, inappropriate use broadly includes the use of these agents to treat clinical conditions that are not caused by bacteria, incorrect choice of drug, incorrect dosage or route of administration, and use for the wrong duration [15]. Even as well-characterized worldwide data are currently lacking, it is estimated that in Organisation for Economic Co-operation and Development countries, as much as 50% of all antimicrobial prescriptions deployed in healthcare can be considered irrational [16]. According to Centers for Disease Control and Prevention (CDC) estimates, inappropriate antimicrobial use in outpatient settings may account for 30–50% of all prescriptions [17,18,19].

Needless to say, making clinical decisions to initiate antimicrobial therapy and drawing appropriate therapeutic regimens in cases of suspected or confirmed infections can be a challenging task. The key points for consideration include the possibility of infection, whether community- or hospital-acquired, suspected causal pathogen, choice of agent based on its spectrum of activity and local resistance patterns, its pharmacokinetic/pharmacodynamic (PK/PD) properties, and possibility of drug interactions. Providing feedback regarding inappropriate prescriptions has been shown to improve rational use of antimicrobials [20,21,22,23]. Currently employed benchmarks on antimicrobial use such as defined daily doses and days of therapy incorporate overall drug use without considerations regarding appropriate prescribing in individual clinical situations. Traditionally assessing optimal or appropriate use remains a challenge in HICs as well as resource-constrained settings. In developed-world settings, the prescribers may be aided by several clinical decision support systems that assist antimicrobial prescribing based on preset rules and are usually integrated with electronic health records [24]. In contrast, the situation in LMICs requires special attention with high patient–provider ratios and lack of adequate contact time, as well as rather limited choice of antimicrobials based on availability [25,26]. Availability of a readily applicable tool to assess rationality of antimicrobial prescriptions is therefore expected to assist in identifying the major problem areas to target and provide accurate feedback in a timely manner. It will also be helpful in assessing the impact of educational and other antimicrobial stewardship (AMS) interventions. We describe a study designed to develop and validate a tool for standardizing the assessment of appropriateness of antimicrobial prescribing for hospitalized patients especially in resource-constrained settings.

## 2. Methodology

The present study was a retrospective observational study conducted at Postgraduate Institute of Medical Education and Research at Chandigarh, one of the largest tertiary care academic healthcare facilities of North India equipped with nearly 2000 beds. The institute has an active AMS program where prospective audit and feedback (PAF) was one of the earliest interventions to be implemented [27]. For PAF, a paper form is used for capturing the relevant data—demographic, diagnosis, comorbidities, prescription, diagnostics, culture, and sensitivity in adequate details. For the present study, the data collected on these paper forms by the AMS personnel as part of routine PAF activities were used. The study was conducted from July to December 2020. The eligibility criteria for the forms to be included for current evaluation included that the patient must have received antimicrobials for at least three days for the management of a suspected/documented infection. Patients receiving antimicrobials as part of an ongoing research study or clinical trial were excluded. The patients were also excluded if one of the study investigators was also the prescriber for that case. The patient data were anonymized before their evaluation by individual raters and experts. 

### 2.1. Development of Antimicrobial Rational Assessment Tool (AmRAT)

An algorithm for assessment of rationality of antimicrobial prescriptions was developed by a multidisciplinary team consisting of intensivists, internal medicine practitioners, clinical pharmacologists, and infectious disease experts. To date, a standardized consensus definition of appropriate use of these agents is lacking. Therefore, a thorough literature search was performed to shortlist the critical elements that can be clearly and unambiguously identified by individual raters to arrive at a decision regarding rational antimicrobial use. Our definitions of appropriateness of antimicrobial therapy were based on criteria suggested by Kunin et al. (1973) [28], CDC worksheets for assessment of appropriateness of antimicrobials [29], United Kingdom National Health Service—Hospital Antimicrobial Prudent Prescribing Indicator (HAPPI) tool [30], and elements defining responsible antibiotic use suggested by Monnier et al. [31]. 

The initial version of the tool was drafted by AKK and NSK based on these resources. Subsequently, a Google form for rationality assessment was created and a set of randomly selected 21 PAF forms were sent to investigators (NS, RM, NG, and NK) for evaluation by the method suggested by Kunin et al. [28]. The experts included in the team comprised an emergency medicine specialist, a critical care consultant, and clinical pharmacologists. The members of the team have been closely involved with the prospective audit and feedback activities of the AMS program of the institute. The categories for rationality assessment were as follows: 

A: Agree with the use of antimicrobials; dosing regimen is appropriate.

B: Agree with use of antimicrobials; dosing regimen is probably appropriate.

C: Agree with use of antimicrobials; a different agent is preferable.

D: Agree with use of antimicrobials; an alternate dose is preferable.

V: Disagree with use of antimicrobials.

The aim of this exercise was to enable subject experts to carefully evaluate the nuances of antimicrobial drug use in individual patients and consider various forms of irrational drug use that may be prevalent in clinical practice.

**Figure 1:**
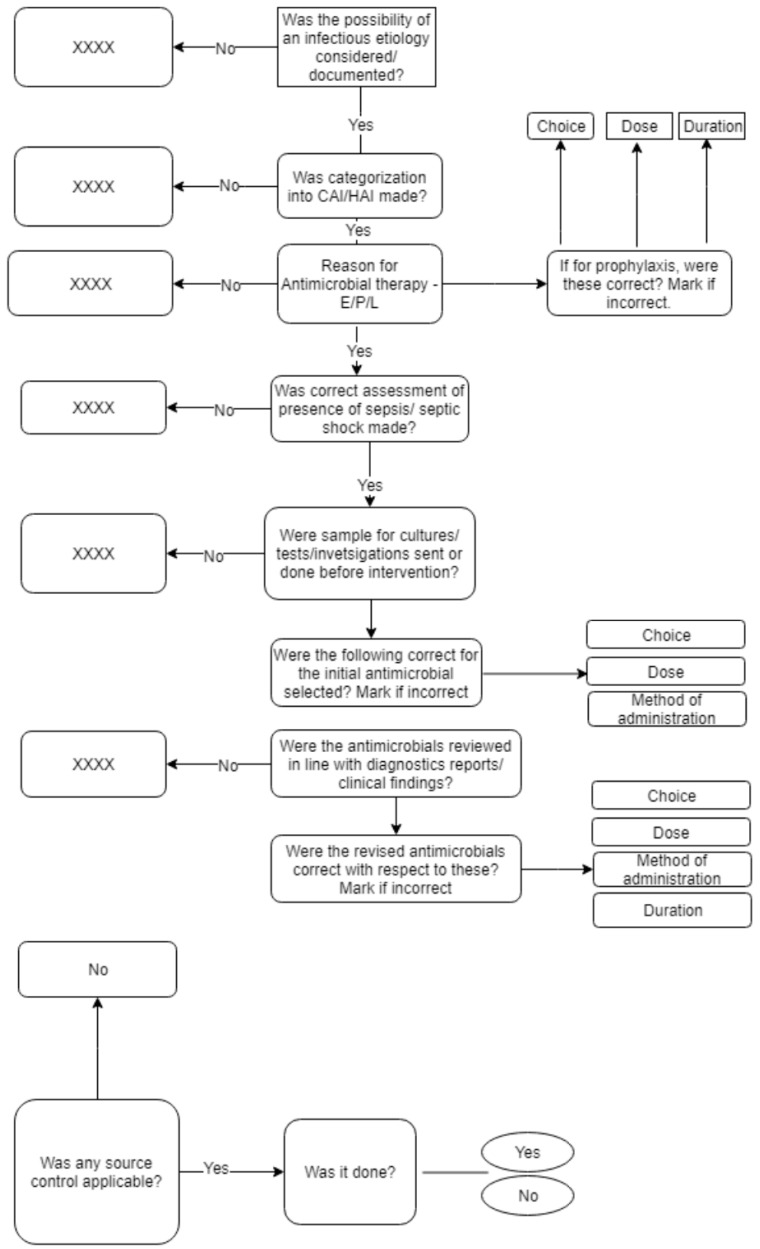
Antimicrobial Rationality Assessment Tool (AmRAT). CAI: Community-acquired infection; HAI: Hospital-acquired infection; E: Empiric; P: Prophylactic; L: Laboratory-based; XXXX—consider as irrational.

This was followed by a meeting where the initial version of the AmRAT tool was discussed amongst the study investigators. Based on consensus opinion, several modifications in the original tool were made including whether categorization into community-acquired/hospital-acquired infection was made, whether the reason for antimicrobial use was categorized as empiric, prophylactic, or laboratory based, correct assessment of sepsis/septic shock, applicability of source control, and assessment of rationality of individual antimicrobial in terms of choice of agent, dose, duration, as well as method of administration. The tool was finalized at the end of the consensus meeting (Figure 1) and was used for further evaluation by individual raters during the study.

### 2.2. Piloting the AmRAT

The following data were evaluated for each patient from the prefilled AMS-PAF forms: demographics, hospital admission and discharge dates, history of illness, comorbidities, antimicrobial treatment before hospitalization, antimicrobial treatment—start/stop dates, indication in terms of empiric/prophylactic or laboratory bases, dosing regimen, reasons for using the specific antimicrobial, specimen sent for microbiological analysis including culture and susceptibility data, any other diagnostic tests of relevance for suspected diagnosis, and routine clinical laboratory investigations.

Among the available completed PAF forms, for the purpose of this pilot study, a set of 30 forms were randomly selected. From each form, one antimicrobial was selected randomly keeping in mind the most frequently prescribed antimicrobials based on our previous point prevalence surveys (PPS) [3].

Thereafter, identical sets of selected forms were evaluated by three groups of raters: group A: Master of Science (M.Sc) (Pharmacology) (*n* = 3), group B: Doctor of Medicine (MD) (Pharmacology) residents (*n* = 3), and group C: Doctor of Medicine with clinical pharmacology specialization—DM (Clinical Pharmacology) residents (*n* = 4) using the final AmRAT tool. These investigators were selected based on their educational backgrounds and years of experience in the prospective audit and feedback activity of the institute. However, all of them had been participating in the institute AMS program and were well versed with the different components of the program operating in the institute. The individual raters were asked to rate the antimicrobial prescriptions into rational and irrational. For each of the antimicrobials, the prescription was considered for the following: choice, dose, and duration of treatment. If the raters considered any of these inappropriate, the prescription was classified as irrational. Further, they were asked to list the reasons for selecting the medicine use as irrational. 

The study experts were also asked to rate the selected 30 prescriptions as rational or irrational and two meetings were organized to arrive at a consensus regarding appropriateness of individual prescriptions. The appropriateness was assessed based upon local epidemiology, previous use of antimicrobials, comorbid illnesses, results of microbiological investigations, patient’s renal function and clinical condition, and standard guidelines such as the Sanford Guide to Antimicrobial Therapy [32], UpToDate [33], and our institutional antimicrobial policy for management of infections. The latter was used to evaluate dose, frequency, and duration of antimicrobial therapy and were adapted according to patient’s body weight and renal clearance. The treatment was deemed to be rational if it met all the requirements as per applicable guidelines and otherwise as irrational and reasons for the same were documented. Any differences between expert ratings were resolved by consensus or voting wherever required. NSK was the moderator for these meetings and did not participate in the voting process. 

### 2.3. Statistical Analysis

Since the study was of an exploratory nature and involved the piloting phase of development of a novel tool, formal sample size calculations were not performed. Interobserver agreement was evaluated using percent raw agreement and Fleiss–Cuzick extension of Cohen’s kappa (κ) statistic for choice of antimicrobial, dose, duration, and overall rationality, respectively. Kappa value with 95% confidence interval was reported. The 95% confidence interval for Cohen’s kappa was calculated using the bootstrap method [34,35]. Appropriateness assessments by study raters were compared with expert consensus opinion, which served as gold standard. Values of kappa were interpreted as per the Landis and Koch protocol (1977) [36]. Kappa values <0 indicated no agreement, 0 to 0.2 as slight agreement, 0.21–0.4 as fair agreement, 0.41–0.6 as moderate agreement, 0.61–0.8 as substantial agreement, and 0.81–1 as near perfect agreement. The sensitivity and specificity estimates were determined comparing assessments of all study raters against the chosen gold standard. All analysis was done using Stata version 16 (College Station, Texas, USA) [37].

## 3. Results

The study raters belonged to three groups that differed from each other in terms of medical background as well as years of experience, although all the participants were familiar with the practice of AMS activities. The group A students had a non-medical background with mean (SD) experience of 4.3 ± 0.9 years. The group B residents had a medical background (MBBS) and AMS experience of three years, while group C residents had a medical background as well as training in clinical pharmacology with a mean (SD) experience of 3.1 ± 2.4 years. 

According to the expert panel, 16 prescriptions were classified as rational while the remaining 14 were classified as irrational. Among the reasons for being adjudicated as irrational, the most common was incorrect dose of antimicrobial in 50% of cases (*n* = 7). The choice of antimicrobial and the duration of administration were found to be incorrect by the expert panel in 42.8% (*n* = 6 each) of prescriptions classified as “irrational”. 

The mean percent agreement for the choice of antimicrobial was over 80% for all three groups of raters when compared to expert group judgment (Table 1). The kappa values were, however, variable, ranging from fair to substantial across the three groups. For the assessment of appropriateness of dose, the mean percentage agreement with expert opinion was found to be 81.1%, 85.6%, and 85.8% for group A, B, and C, respectively. Kappa values ranged from fair to moderate in group A, and moderate to substantial among group B and C raters. High mean percent agreement was found for the duration of antimicrobial administration as well (88%, 85.1%, and 86.7% for group A, B, and C, respectively), although the kappa values were similarly variable. For the overall assessment of rationality, the mean percentage agreement with expert group judgment was 76.7% for group A, 68.9% for group B, and 77.5% for Group C. The kappa values indicated moderate agreement for all raters in group A, and fair to moderate in group B as well as group C.

Comparing the raters within each group, we found moderate percentage agreements over choice of antimicrobial for all three groups of raters (Table 2). However, considerable agreements were noted for dose (above 75%) as well as duration of therapy (above 80%). Percent agreements for the three groups were 62.2% for group A, 55.6% for group B, and 66.1% for group C for the overall assessment of rationality. Kappa coefficients were, however, lower across all categories of raters. Interobserver agreement between group A raters was found to be “slight” for choice of antimicrobial (k = 0.19) and dose (k = 0.15), “fair” for overall rationality (k = 0.21), while moderate agreement was found for duration of administration (k = 0.60). For group B, there was “fair agreement” for the choice of antimicrobial (k = 0.31) as well as dose (k = 0.38) and moderate agreement was noted for duration of antimicrobial administration (k = 0.59). However, the agreement for overall adjudication of rationality was found to be “slight” (k = 0.11). Similarly, among group C raters, there was slight agreement for choice of agent, fair agreement for dose, and substantial agreement for the duration of administration. However, for the overall classification of rationality, a “fair” agreement was found within the raters (k = 0.30). 

The overall estimates of sensitivity and specificity for rationality assessment were 80% and 68.6%, respectively (Table 3), indicating consistent assessments by the study raters when compared to the expert opinion which was taken as gold standard. The sensitivity, specificity, and correctly classified assessments for each of the study raters when compared to expert opinion as gold standard are detailed in Table S1.

## 4. Discussion

We developed an algorithm to assess rationality of antimicrobial prescriptions and piloted it for use among AMS personnel with diverse educational backgrounds and duration of clinical experience. The principal decision points in the algorithm include the categorization into community-acquired or hospital-acquired infections, documentation of reason for starting antimicrobial therapy, consideration of possibility of sepsis, sending microbiological diagnostic investigations before starting chemotherapy, timely review of prescription based on diagnostic results, implementation of source control, and choice of antimicrobial including its dose, duration, and method of administration. In the absence of a well-established gold standard tool for the assessment of antimicrobial rationality, we used expert consensus opinion as the standard for comparison with individual raters’ evaluations. The percentage agreement between expert assessments and AMS personnel for the overall assessment of rationality of prescriptions was found to be 70 or above for 9 out of 10 raters. Based on kappa statistic, the agreement between raters and experts ranged from fair to moderate, the latter being in 70% of study personnel. For the individual components of choice, dose, and duration of antimicrobial therapy, the average agreement was above 80% in all three categories of raters. The kappa values for choice of antimicrobial as well as dose of selected agent ranged from fair to substantial. For the selection of agent, this was somewhat expected because the decision is often subjective as has been demonstrated in previous studies where even infectious disease experts who were well aware of local resistance patterns and patients’ profiles demonstrated considerable disagreement with respect to antimicrobial choices [38,39]. However, agreement over dose varied considerably and these findings are somewhat unexpected. The consensus guidelines for rational antimicrobial prescribing have been recently introduced in our institute and we hope the disagreement over dose should substantially improve over time and will be a subject of future investigations. Among the various criteria of rationality assessed in this study, agreement over appropriateness of duration of antimicrobial treatment was found to be highest with kappa agreement coefficients ranging from “moderate” to “almost perfect”. As highlighted above, while irrational antimicrobial use is high in resource-constrained settings, the situation is further exacerbated by lack of adequate diagnostic tools to guide clinicians in safely de-escalating antimicrobial therapy [40]. For our AMS program interventions, special emphasis is laid upon antimicrobial de-escalation and all the AMS personnel are trained to provide timely feedback on antimicrobial discontinuation or switch to a narrow-spectrum agent wherever indicated. We also documented substantial within-group inter-rater variability in our study for all three categories of raters for the interpretation of choice, dose, and duration of antimicrobial therapy, as well as for the overall assessment of rationality. 

Given the lack of universal agreement on what constitutes appropriate antimicrobial therapy, previous studies have aimed to assess rationality based on various diverse definitions. These include prescribing in accordance with international guidelines or local protocols, expert opinions, in vitro susceptibilities, and computerized algorithms, amongst others, as well as a combination of these methods [38,41,42,43,44,45,46,47]. The principal purpose of developing AmRAT for resource-limited settings was to allow rapid assessment of rationality of antimicrobial prescriptions that could be carried out by a wide array of AMS personnel with different educational backgrounds and varying degrees of experience. Traditionally at our facility, we have been practicing a “two-step” prospective audit and feedback service as part of AMS activities [27]. For this, a preliminary screening of individual cases from wards or intensive care units is carried out by a visiting AMS team member. Thereafter, these cases are presented to a senior consultant/team member who assesses the rationality and conveys the suggestions for therapy modification or cessation to the concerned clinician through written or telephonic communication. This is a rather intensive exercise which was considered necessary while the AMS program was in its nascent stages. As a result, the services could only be extended to few wards and critical care units of the hospital. However, for enabling expansion of services to other underserved areas, it is critical that a rapid assessment of antimicrobial prescriptions is carried out so that a timely feedback for the treating physicians could be ensured. Given the significantly high patient–provider ratios in the LMIC settings and often the lack of interest or availability of trained infectious disease physicians, the role of clinical/medical pharmacologists, microbiologists, medical graduates, public health specialists, and healthcare professionals from various allied disciplines is deemed essential if AMS programs are to be successful in resource-limited settings. An algorithm that incorporates all the elements deemed essential for a rational antimicrobial prescription was therefore developed in consultation with experienced subject experts to allow bedside assessment of appropriateness and feedback by observers from diverse backgrounds. We found more than 68% mean agreement between study raters using AmRAT and expert opinion for appropriateness of choice of agent, its dose and duration, as well as overall assessment of rationality of included study prescriptions. Although percentage agreements were high, the kappa values were low in our study, reflecting the classic paradox associated with kappa coefficients wherein high percent agreements may be seen associated with low kappa values [48,49]. As measures of agreement among raters, both percentage agreement and kappa have their strengths and weaknesses [50]. While percentage agreement is easy to calculate and interpret, it does not account for chance agreement. The kappa statistic, on the other hand, accounts for the possibility of guessing by raters, but the underlying assumptions may not be well supported, and can potentially lower the estimates of agreement considerably. Since our study raters were well trained and had at least three years of mean AMS experience and were unlikely to be guessing rationality, percent agreement could be considered a reliable measure of inter-rater agreement for our rationality assessment algorithm. Moreover, taking expert opinion as gold standard in the absence of universally accepted definitions of rationality, our tool demonstrated good sensitivity and specificity. In the presence of a reference standard, sensitivity and specificity can be useful estimates of validity rather than simple kappa coefficients.

One of the strengths of our study is that we developed a criteria-based algorithm for assessment of rationality of antimicrobial prescriptions based on survey of available resources as well as inputs from a multidisciplinary team experienced in the management of infectious diseases. In this pilot study, the tool was evaluated by a team of raters with diverse educational backgrounds and varying degrees of antimicrobial stewardship experience and high degree of percentage agreement among them could be documented. Limitations of our study include the utilization of AMS forms for assessment of rationality instead of real-time clinical evaluation and consultation with the treating physicians. However, use of such data forms reflects the real-world AMS practice and is usually deemed adequate for the assessment of several key aspects of rationality of antimicrobial prescriptions. We attempted to minimize this bias by encouraging evaluation of all the patient data available in the form of written notes of AMS personnel, but since the forms were anonymized, it was not possible to contact individual prescribers during the study. This was a pilot study where the tool was evaluated by 10 raters, all belonging to the same institution. In addition, the evaluations were based on a limited number of prescriptions that were randomly selected and rationality of only one chosen antimicrobial was assessed from each of the prescriptions. To obtain more generalizable results and to test applicability in diverse clinical settings, a larger study is warranted. We plan to evaluate this tool in all centers that are affiliated with our center as part of Indian Council of Medical Research’s grant for Advanced Center of Clinical Pharmacology for Antimicrobial Stewardship and research on antimicrobial usage. The applicability of our tool to more complex clinical situations, where the possibility of drug interactions and double antimicrobial cover exists, also needs to be evaluated in future multicentric studies.

## Figures and Tables

**Table 1:** Percentage of agreement and kappa (95% CI) of each observer with expert.

	Observer	Agreement	Kappa (95% CI)
	***Choice of antimicrobial***		
**Group A**	**1**	93.3%	0.79 (0.50,1.00)
	**2**	76.7%	0.38 (−0.02,0.78)
	**3**	73.3%	0.4 (0.03,0.77)
	**Mean**	81.1%	
**Group B**	**1**	86.7%	0.66 (0.33,0.98)
	**2**	76.7%	0.46 (0.10,0.82)
	**3**	80.0%	0.28 (−0.18,0.74)
	**Mean**	81.1%	
**Group C**	**1**	83.3%	0.56 (0.19,0.92)
	**2**	86.7%	0.66 (0.33,0.98)
	**3**	76.7%	0.31 (−0.11,0.74)
	**4**	80.0%	0.38 (−0.06,0.81)
	**Mean**	81.7%	
	***Dose***		
**Group A**	**1**	83.3%	0.35 (−0.13,0.83)
	**2**	80.0%	0.28 (−0.18,0.74)
	**3**	80.0%	0.49 (0.11,0.86)
	**Mean**	81.1%	
**Group B**	**1**	86.7%	0.66 (0.33,0.98)
	**2**	80.0%	0.52 (0.17,0.88)
	**3**	90.0%	0.67 (0.30,1.00)
	**Mean**	85.6%	
**Group C**	**1**	86.7%	0.63 (0.27,0.98)
	**2**	86.7%	0.63 (0.27,0.98)
	**3**	83.3%	0.51 (0.11,0.91)
	**4**	86.7%	0.58 (0.19,0.97)
	**Mean**	85.8%	
	***Duration***		
**Group A**	**1**	79.0%	0.5 (0.02,0.98)
	**2**	87.5%	0.73 (0.28,1)
	**3**	100.0%	1.00 (0.72,1.00)
	**Mean**	88.0%	
**Group B**	**1**	81.2 %	0.56 (0.03,1.00)
	**2**	87.5%	0.73 (0.28,1.00)
	**3**	86.7%	0.68 (0.17,1.00)
	**Mean**	85.1%	
**Group C**	**1**	76.5%	0.48 (−0.03,0.99)
	**2**	81.3%	0.57 (0.05,1.00)
	**3**	100.0%	1.00 (0.65,1.00)
	**4**	92.9%	0.84 (0.41,1.00)
	**Mean**	86.7%	
	***Overall rationality***		
**Group A**	**1**	80.0%	0.57 (0.25,0.89)
	**2**	76.7%	0.52 (0.19,0.85)
	**3**	73.3%	0.47 (0.13,0.80)
	**Mean**	76.7%	
**Group B**	**1**	73.3%	0.46 (0.13,0.80)
	**2**	63.3%	0.22 (−0.15,0.60)
	**3**	70.0%	0.34 (−0.03,0.71)
	**Mean**	68.9%	
**Group C**	**1**	80.0%	0.60 (0.29,0.90)
	**2**	80.0%	0.60 (0.29,0.90)
	**3**	80.0%	0.59 (0.28,0.90)
	**4**	70.0%	0.37 (0.00,0.73)
	**Mean**	77.5%	

Group A: M.Sc. (Pharmacology); Group B: MD residents; Group C: DM (Clinical Pharmacology) residents.

**Table 2:** Group-specific inter-rater reliability coefficients.

	% Agreement (95% CI)	Kappa (95% CI)
**Group A**		
*Choice*	64.4 (51.8,77.1)	0.19 (−0.1,0.47)
*Dose*	77.8 (65.8,89.7)	0.15 (−0.03,0.33)
*Duration*	81.3 (56.2,100)	0.60 (0.2,1.00)
*Overall rationality*	62.2 (49.7,74.8)	0.21 (−0.06,0.47)
**Group B**		
*Choice*	71.1 (58.6,83.7)	0.31 (0.04,0.59)
*Dose*	75.6 (63.4,87.8)	0.38 (0.09,0.66)
*Duration*	81.3 (60.1,100)	0.59 (0.2,0.99)
*Overall rationality*	55.6 (43.6,67.5)	0.11 (−0.13,0.35)
**Group C**		
*Choice*	68.9 (58.3,79.5)	0.20 (−0.01,0.42)
*Dose*	79.4 (69,89.9)	0.38 (0.14,0.61)
*Duration*	82.3 (61.7,100)	0.62 (0.27,0.98)
*Overall rationality*	66.1 (55.3,76.9)	0.30 (0.09,0.51)

Group A: M.Sc. (Pharmacology); Group B: MD residents; Group C: DM (Clinical Pharmacology) residents.

**Table 3:** Overall sensitivity and specificity estimates compared to gold standard.

	Percent	95% CI	
***Choice of Antimicrobial***			
Sensitivity	82.5%	77.1%	87.1%
Specificity	76.7%	64.0%	86.6%
***Dose***			
Sensitivity	91.3%	86.9%	94.6%
Specificity	61.4%	49.0%	72.8%
***Duration***			
Sensitivity	92.4%	84.9%	96.9%
Specificity	78.5%	66.5%	87.7%
***Overall Rationality***			
Sensitivity	80.00%	73.00%	85.90%
Specificity	68.60%	60.20%	76.10%

## Data Availability

Data is contained within the article or supplementary material.

## Supplementary Materials

The following are available online at https://www.mdpi.com/2079-6382/10/2/200/s1, Table S1: Sensitivity, specificity, and correctly classified (expert as gold standard).
