# Supplementary material for: Assessment of Appropriateness of Antimicrobial Therapy in Resource-Constrained Settings: Development and Piloting of a Novel Tool—AmRAT
Source: Antibiotics (Basel). 2021 Feb 19;10(2):200. doi: 10.3390/antibiotics10020200 (PMC7923130; doi:10.3390/antibiotics10020200)
Supplement: Supplementary file 1 [file antibiotics-10-00200-s001.pdf]

Supplementary Table S1: Sensitivity, specificity and correctly classified (Expert as Gold standard)

Choice of antimicrobial		Sensitivity	Specificity	Correctly classified
Group A	1	96%	83%	93%
	2	79%	67%	77%
	3	67%	100%	73%
Group B	1	83%	100%	87%
	2	71%	100%	77%
	3	92%	33%	80%
Group C	1	83%	83%	83%
	2	83%	100%	87%
	3	83%	50%	77%
	4	88%	50%	80%
Dose				
Group A	1	100%	29%	83%
	2	96%	29%	80%
	3	83%	71%	80%
Group B	1	87%	86%	87%
	2	78%	86%	80%
	3	100%	57%	90%
Group C	1	91%	71%	87%
	2	91%	71%	87%
	3	91%	57%	83%
	4	96%	57%	87%
Duration				
Group A	1	92%	57%	79%
	2	89%	86%	88%
	3	100%	100%	100%
Group B	1	89%	71%	81%
	2	89%	86%	88%
	3	100%	71%	87%
Group C	1	80%	71%	76%
	2	89%	71%	81%
	3	100%	100%	100%
	4	100%	80%	93%
Overall rationality				
Group A	1	100%	57%	80%
	2	88%	64%	77%
	3	69%	79%	73%

**Group B**	**1**	75%	71%	73%
	**2**	38%	93%	63%
	**3**	94%	43%	70%
**Group C**	**1**	81%	79%	80%
	**2**	81%	79%	80%
	**3**	88%	71%	80%
	**4**	88%	50%	70%

Group A: M.Sc. (Pharmacology); Group B: MD residents; Group C: DM (Clinical Pharmacology)residents
